# 1-Methyl-2,3-dihydro-1*H*-benzimidazole-2-selone

**DOI:** 10.1107/S1600536812013700

**Published:** 2012-04-13

**Authors:** Gunay Z. Mammadova, Zhanna V. Matsulevich, Vladimir K. Osmanov, Alexander V. Borisov, Victor N. Khrustalev

**Affiliations:** a Baku State University, Z. Khalilov St 23, Baku AZ-1148, Azerbaijan; b R.E. Alekseev Nizhny Novgorod State Technical University, 24 Minin St, Nizhny Novgorod, 603950, Russian Federation; c X-Ray Structural Centre, A.N. Nesmeyanov Institute of Organoelement Compounds, Russian Academy of Sciences, 28 Vavilov St, B-334, Moscow 119991, Russian Federation

## Abstract

The title compound C_8_H_8_N_2_Se, is the product of the reaction of 2-chloro-1-methyl­benzimidazole with sodium hydro­selenide. The mol­ecule is almost planar (r.m.s. deviation = 0.041 Å) owing to the presence of the long chain of conjugated bonds (Se=C—NMe—C=C—C=C—C=C—NH). The C=Se bond length [1.838 (2) Å] corresponds well to those found in the close analogs and indicates its pronounced double-bond character. In the crystal, mol­ecules form helicoidal chains along the *b* axis by means of N—H⋯Se hydrogen bonds.

## Related literature

For selones as potential anti­thyroid drugs, see: Taurog *et al.* (1994 ▶); Roy & Mugesh (2005 ▶, 2006 ▶); Roy *et al.* (2007 ▶, 2011 ▶). For related compounds, see: Guziec & Guziec (1994 ▶); Husebye *et al.* (1997 ▶); Aydin *et al.* (1999 ▶); Akkurt *et al.* (2004 ▶, 2011 ▶); Landry *et al.* (2006 ▶); Nakanishi *et al.* (2008 ▶); Mammadova *et al.* (2011 ▶). For hypervalent adducts of selones with dihalogens and inter­halogens, see: Aragoni *et al.* (2001 ▶); Boyle & Godfrey (2001 ▶); Roy *et al.* (2011 ▶).
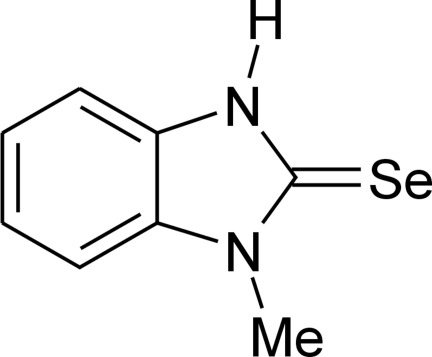


## Experimental

### 

#### Crystal data

C_8_H_8_N_2_Se
*M* *_r_* = 211.12
Monoclinic, 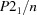

*a* = 9.9434 (13) Å
*b* = 5.8472 (8) Å
*c* = 13.6387 (18) Å
β = 95.360 (2)°
*V* = 789.50 (18) Å^3^
*Z* = 4
Mo *K*α radiation
μ = 4.69 mm^−1^
*T* = 100 K
0.24 × 0.20 × 0.20 mm

#### Data collection

Bruker APEXII CCD diffractometer
Absorption correction: multi-scan (*SADABS*; Sheldrick, 2003 ▶) *T*_min_ = 0.399, *T*_max_ = 0.454
9470 measured reflections
2304 independent reflections
1941 reflections with *I* > 2σ(*I*)
*R*_int_ = 0.030

#### Refinement

*R*[*F* ^2^ > 2σ(*F* ^2^)] = 0.027
*wR*(*F* ^2^) = 0.068
*S* = 1.00
2304 reflections
101 parameters
H-atom parameters constrained
Δρ_max_ = 1.10 e Å^−3^
Δρ_min_ = −0.28 e Å^−3^

### 

Data collection: *APEX2* (Bruker, 2005 ▶); cell refinement: *SAINT-Plus* (Bruker, 2001 ▶); data reduction: *SAINT-Plus*; program(s) used to solve structure: *SHELXTL* (Sheldrick, 2008 ▶); program(s) used to refine structure: *SHELXTL*; molecular graphics: *SHELXTL*; software used to prepare material for publication: *SHELXTL*.

## Supplementary Material

Crystal structure: contains datablock(s) global, I. DOI: 10.1107/S1600536812013700/rk2347sup1.cif

Structure factors: contains datablock(s) I. DOI: 10.1107/S1600536812013700/rk2347Isup2.hkl

Supplementary material file. DOI: 10.1107/S1600536812013700/rk2347Isup3.cml

Additional supplementary materials:  crystallographic information; 3D view; checkCIF report

## Figures and Tables

**Table 1:** Hydrogen-bond geometry (Å, °)

*D*—H⋯*A*	*D*—H	H⋯*A*	*D*⋯*A*	*D*—H⋯*A*
N3—H3*N*⋯Se1^i^	0.91	2.58	3.471 (2)	168

Symmetry code: (i) 
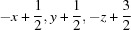
.

**Fig. 1.**
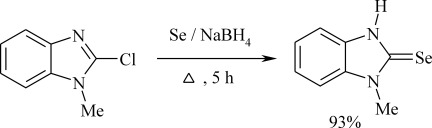
Reaction of 2-chloro-1-methylbenzimidazole with sodium hydroselenide.

**Fig. 2.**
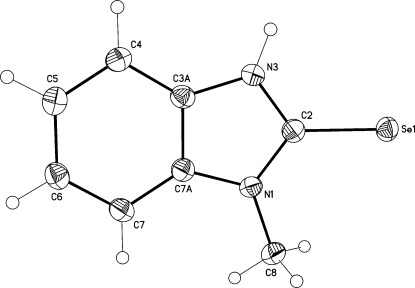
Molecular structure of I with the atom numbering scheme. Displacement ellipsoids are shown at the 50% probability level. H atoms are presented as a small spheres of arbitrary radius.

**Fig. 3.**
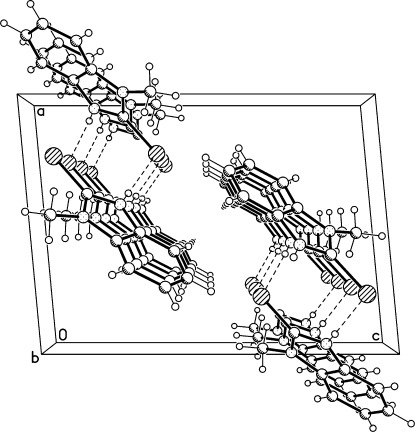
The helicoidal chains of I along the b axis. Dashed lines indicate the intermolecular N–H···Se hydrogen bonds.

## supplementary crystallographic information

### Comment

In the last years, the selone derivatives have attracted considerable attention owing to their antithyroid properties (Taurog *et al.*, 1994; Roy & Mugesh, 2005, 2006; Roy *et al.*, 2007, 2011) as well as selone-selenol tautomerism (Guziec & Guziec, 1994; Husebye *et al.*, 1997; Landry *et al.*, 2006; Mammadova *et al.*, 2011). Moreover, they are used as substrates in the preparation of hypervalent adducts in reactions with dihalogens and interhalogens (Aydin *et al.*, 1999; Aragoni *et al.*, 2001; Boyle & Godfrey, 2001; Akkurt *et al.*, 2004, 2011; Roy *et al.*, 2011).

The title compound - 1-methyl-2,3-dihydro-1*H*-benzimidazole-2-selone, I was obtained by a reaction of 2-chloro-1-methylbenzimidazole with sodium hydroselenide (Fig. 1). The molecule of I is almost planar (r.m.s. deviation = 0.041Å) owing to the presence of the long chain of conjugated bonds (Se═C—NMe—C═C—C═C—C═C—NH) (Fig. 2). The length of the C═Se bond (1.838 (2)Å) corresponds well to those found in the closer analogs of I - 1,3-dimethylbenzimidazole-2-selone (1.825 (7)Å) (Aydin *et al.*, 1999), 1-ethyl-3-(2-phenylethyl)benzimidazole-2-selone (1.829 (3)Å) (Akkurt *et al.*, 2004) and 1,3-bis(3-phenylpropyl)-1*H*-1,3-benzimidazole-2(3*H*)-selone (1.828 (2)Å) (Akkurt *et al.*, 2011) indicating its pronounced double character.

In the crystal, molecules form helicoidal chains along the *b* axis by means of intermolecular N—H···Se^i^ hydrogen bonds (Fig. 3, Table 1). Symmetry code: (i) -*x*+1/2, *y*+1/2, -*z*+3/2.

### Experimental

A solution of NaBH_4_ (3.83 g, 100.0 mmol) in water (25 ml) was added to a suspension of selenium (3.77 g, 47.7 mmol) in water (30 ml) with stirring at room temperature under argon. After 15 min, a solution of 2-chloro-1-methylbenzimidazole (6.60 g, 39.6 mmol) in C_2_H_5_OH (25 ml) was added. The resulting mixture was refluxed for 5 h. At the end of the reaction the solvents were evaporated *in vacuo*, and formed precipitate was extracted with CH_2_Cl_2_. Then the extract was dried over MgSO_4_. Further crystallization from CH_2_Cl_2_ gives the selone I as colourless crystals. Yield is 7.81 g (93%). *M*.p. = 470-471 K. IR(KBr), ν/cm^-1^: 3095, 1618, 1438, 1382, 11330, 1223, 1091, 746, 709. ^1^H NMR (*DMSO*-*d*_6_, 600 MHz, 303 K): δ = 3.63 (s, 3H, *Me*), 7.15 (t, 1H, H6, *J* = 7.1), 7.19 (t, 1H, H5, *J* = 7.1), 7.37 (d, 1H, H4, *J* = 7.1), 7.43 (d, 1H, H7, *J* = 7.1), 13.25 (s, 1H, H3). Anal. Calc. for C_8_H_8_N_2_Se: C, 45.53; H, 3.82; N, 13.27. Found: C, 45.43; H, 2.78; N, 13.19.

### Refinement

The hydrogen atom of the amino group was localized in the difference-Fourier map and included in the refinement with fixed positional and isotropic displacement parameters [*U*_iso_(H) = 1.2*U*_eq_(N)]. The other hydrogen atoms were placed in calculated positions with C—H = 0.95–0.98Å and refined in the riding model with fixed isotropic displacement parameters [*U*_iso_(H) = 1.5*U*_eq_(C) for the methyl group and 1.2*U*_eq_(C) for the other groups].

### Crystal data

**Table:**

C_8_H_8_N_2_Se	*F*(000) = 416
*M**_r_* = 211.12	*D*_x_ = 1.776 Mg m^−^^3^
Monoclinic, *P*2_1_/*n*	Mo *K*α radiation, λ = 0.71073 Å
Hall symbol: -P 2yn	Cell parameters from 3916 reflections
*a* = 9.9434 (13) Å	θ = 2.4–32.5°
*b* = 5.8472 (8) Å	µ = 4.69 mm^−^^1^
*c* = 13.6387 (18) Å	*T* = 100 K
β = 95.360 (2)°	Prism, yellow
*V* = 789.50 (18) Å^3^	0.24 × 0.20 × 0.20 mm
*Z* = 4	

### Data collection

**Table:**

Bruker APEXII CCD diffractometer	2304 independent reflections
Radiation source: fine-focus sealed tube	1941 reflections with *I* > 2σ(*I*)
Graphite monochromator	*R*_int_ = 0.030
φ– and ω–scans	θ_max_ = 30.0°, θ_min_ = 2.4°
Absorption correction: multi-scan (*SADABS*; Sheldrick, 2003)	*h* = −13→13
*T*_min_ = 0.399, *T*_max_ = 0.454	*k* = −8→8
9470 measured reflections	*l* = −19→19

### Refinement

**Table:**

Refinement on *F*^2^	Primary atom site location: structure-invariant direct methods
Least-squares matrix: full	Secondary atom site location: difference Fourier map
*R*[*F*^2^ > 2σ(*F*^2^)] = 0.027	Hydrogen site location: difference Fourier map
*wR*(*F*^2^) = 0.068	H-atom parameters constrained
*S* = 1.00	*w* = 1/[σ^2^(*F*_o_^2^) + (0.0345*P*)^2^ + 0.745*P*] where *P* = (*F*_o_^2^ + 2*F*_c_^2^)/3
2304 reflections	(Δ/σ)_max_ = 0.001
101 parameters	Δρ_max_ = 1.10 e Å^−^^3^
0 restraints	Δρ_min_ = −0.28 e Å^−^^3^

### Special details

**Table:**

Geometry. All s.u.'s (except the s.u. in the dihedral angle between two l.s. planes) are estimated using the full covariance matrix. The cell s.u.'s are taken into account individually in the estimation of s.u.'s in distances, angles and torsion angles; correlations between s.u.'s in cell parameters are only used when they are defined by crystal symmetry. An approximate (isotropic) treatment of cell s.u.'s is used for estimating s.u.'s involving l.s. planes.
Refinement. Refinement of *F*^2^ against ALL reflections. The weighted *R*-factor *wR* and goodness of fit *S* are based on *F*^2^, conventional *R*-factors *R* are based on *F*, with *F* set to zero for negative *F*^2^. The threshold expression of *F*^2^ > σ(*F*^2^) is used only for calculating *R*-factors(gt) *etc*. and is not relevant to the choice of reflections for refinement. *R*-factors based on *F*^2^ are statistically about twice as large as those based on *F*, and *R*-factors based on ALL data will be even larger.

### Fractional atomic coordinates and isotropic or equivalent isotropic displacement parameters (Å^2^)

**Table:**

	*x*	*y*	*z*	*U*_iso_*/*U*_eq_	
Se1	0.26128 (2)	1.12016 (4)	0.878637 (16)	0.02213 (7)	
N1	0.47551 (17)	0.8571 (3)	0.80856 (13)	0.0200 (3)	
C2	0.3951 (2)	1.0457 (4)	0.79997 (16)	0.0203 (4)	
N3	0.43002 (18)	1.1660 (3)	0.72130 (13)	0.0209 (3)	
H3N	0.3892	1.2987	0.7005	0.025*	
C3A	0.5294 (2)	1.0505 (4)	0.67611 (16)	0.0206 (4)	
C4	0.5950 (2)	1.0978 (4)	0.59324 (16)	0.0224 (4)	
H4	0.5785	1.2350	0.5568	0.027*	
C5	0.6860 (2)	0.9352 (4)	0.56600 (17)	0.0242 (4)	
H5	0.7320	0.9611	0.5090	0.029*	
C6	0.7122 (2)	0.7339 (4)	0.62006 (16)	0.0240 (4)	
H6	0.7745	0.6260	0.5986	0.029*	
C7	0.6488 (2)	0.6890 (4)	0.70443 (16)	0.0220 (4)	
H7	0.6674	0.5539	0.7419	0.026*	
C7A	0.5571 (2)	0.8506 (3)	0.73148 (15)	0.0200 (4)	
C8	0.4688 (2)	0.6817 (4)	0.88271 (17)	0.0255 (4)	
H8A	0.4578	0.7533	0.9464	0.038*	
H8B	0.5524	0.5919	0.8876	0.038*	
H8C	0.3918	0.5810	0.8644	0.038*	

### Atomic displacement parameters (Å^2^)

**Table:**

	*U*^11^	*U*^22^	*U*^33^	*U*^12^	*U*^13^	*U*^23^
Se1	0.02158 (11)	0.02058 (11)	0.02486 (11)	−0.00119 (8)	0.00548 (7)	−0.00250 (8)
N1	0.0202 (8)	0.0177 (8)	0.0225 (8)	−0.0010 (6)	0.0030 (6)	0.0026 (6)
C2	0.0191 (9)	0.0196 (9)	0.0219 (9)	−0.0018 (7)	0.0011 (7)	−0.0011 (7)
N3	0.0219 (8)	0.0174 (8)	0.0236 (8)	0.0020 (6)	0.0037 (6)	0.0018 (6)
C3A	0.0195 (9)	0.0184 (9)	0.0236 (10)	−0.0006 (7)	0.0007 (7)	0.0000 (7)
C4	0.0227 (9)	0.0221 (10)	0.0222 (9)	−0.0013 (8)	0.0017 (7)	0.0027 (8)
C5	0.0231 (10)	0.0272 (11)	0.0227 (10)	−0.0019 (8)	0.0037 (8)	−0.0019 (8)
C6	0.0204 (10)	0.0242 (10)	0.0274 (11)	0.0021 (8)	0.0029 (8)	−0.0023 (8)
C7	0.0200 (9)	0.0192 (9)	0.0263 (10)	0.0007 (7)	0.0005 (8)	−0.0008 (8)
C7A	0.0184 (9)	0.0199 (9)	0.0216 (9)	−0.0015 (7)	0.0009 (7)	−0.0005 (7)
C8	0.0258 (10)	0.0237 (10)	0.0275 (11)	0.0005 (8)	0.0058 (8)	0.0063 (8)

### Geometric parameters (Å, º)

**Table:**

Se1—C2	1.838 (2)	C4—H4	0.9500
N1—C2	1.361 (3)	C5—C6	1.400 (3)
N1—C7A	1.387 (3)	C5—H5	0.9500
N1—C8	1.446 (3)	C6—C7	1.388 (3)
C2—N3	1.355 (3)	C6—H6	0.9500
N3—C3A	1.388 (3)	C7—C7A	1.387 (3)
N3—H3N	0.9090	C7—H7	0.9500
C3A—C4	1.385 (3)	C8—H8A	0.9800
C3A—C7A	1.405 (3)	C8—H8B	0.9800
C4—C5	1.387 (3)	C8—H8C	0.9800
			
C2—N1—C7A	109.74 (17)	C6—C5—H5	119.0
C2—N1—C8	124.74 (18)	C7—C6—C5	121.3 (2)
C7A—N1—C8	125.35 (18)	C7—C6—H6	119.4
N3—C2—N1	107.24 (18)	C5—C6—H6	119.4
N3—C2—Se1	126.35 (16)	C7A—C7—C6	116.9 (2)
N1—C2—Se1	126.40 (16)	C7A—C7—H7	121.5
C2—N3—C3A	110.20 (18)	C6—C7—H7	121.5
C2—N3—H3N	123.4	C7—C7A—N1	131.8 (2)
C3A—N3—H3N	126.3	C7—C7A—C3A	121.6 (2)
C4—C3A—N3	132.4 (2)	N1—C7A—C3A	106.60 (18)
C4—C3A—C7A	121.4 (2)	N1—C8—H8A	109.5
N3—C3A—C7A	106.12 (18)	N1—C8—H8B	109.5
C3A—C4—C5	116.8 (2)	H8A—C8—H8B	109.5
C3A—C4—H4	121.6	N1—C8—H8C	109.5
C5—C4—H4	121.6	H8A—C8—H8C	109.5
C4—C5—C6	121.9 (2)	H8B—C8—H8C	109.5
C4—C5—H5	119.0		
			
C7A—N1—C2—N3	−3.4 (2)	C5—C6—C7—C7A	1.1 (3)
C8—N1—C2—N3	−178.94 (19)	C6—C7—C7A—N1	178.4 (2)
C7A—N1—C2—Se1	175.35 (15)	C6—C7—C7A—C3A	0.0 (3)
C8—N1—C2—Se1	−0.2 (3)	C2—N1—C7A—C7	−175.6 (2)
N1—C2—N3—C3A	2.5 (2)	C8—N1—C7A—C7	−0.1 (4)
Se1—C2—N3—C3A	−176.20 (15)	C2—N1—C7A—C3A	2.9 (2)
C2—N3—C3A—C4	178.0 (2)	C8—N1—C7A—C3A	178.45 (19)
C2—N3—C3A—C7A	−0.7 (2)	C4—C3A—C7A—C7	−1.5 (3)
N3—C3A—C4—C5	−176.7 (2)	N3—C3A—C7A—C7	177.40 (19)
C7A—C3A—C4—C5	1.9 (3)	C4—C3A—C7A—N1	179.74 (19)
C3A—C4—C5—C6	−0.8 (3)	N3—C3A—C7A—N1	−1.3 (2)
C4—C5—C6—C7	−0.7 (3)		

### Hydrogen-bond geometry (Å, º)

**Table:**

*D*—H···*A*	*D*—H	H···*A*	*D*···*A*	*D*—H···*A*
N3—H3*N*···Se1^i^	0.91	2.58	3.471 (2)	168

Symmetry code: (i) −*x*+1/2, *y*+1/2, −*z*+3/2.
